# Inflammatory corpuscle AIM2 facilitates macrophage foam cell formation by inhibiting cholesterol efflux protein ABCA1

**DOI:** 10.1038/s41598-024-61495-4

**Published:** 2024-05-11

**Authors:** Shujiang Zhuo, Sufei Song, Chaoyi Wang, Zhe Wang, Ming Zhang, Daobin Lin, Kaili Chen

**Affiliations:** 1 Department of Cardiology, Hainan Provincial Hospital of Traditional Chinese Medicine, Haikou, China; 2 Department of Data Science, Macau University of Science and Technology, Macau, China; 3 Department of Traditional Chinese Medicine, Hainan Medical University, Haikou, China

**Keywords:** Inflammatory corpuscle, Atherosclerosis, AIM2, ABCA1, Cholesterol efflux, Cell biology, Computational biology and bioinformatics, Molecular biology, Cardiology, Medical research, Pathogenesis

## Abstract

The inflammatory corpuscle recombinant absents in melanoma 2 (AIM2) and cholesterol efflux protein ATP binding cassette transporter A1(ABCA1) have been reported to play opposing roles in atherosclerosis (AS) plaques. However, the relationship between AIM2 and ABCA1 remains unclear. In this study, we explored the potential connection between AIM2 and ABCA1 in the modulation of AS by bioinformatic analysis combined with in vitro experiments. The GEO database was used to obtain AS transcriptional profiling data; screen differentially expressed genes (DEGs) and construct a weighted gene co-expression network analysis (WGCNA) to obtain AS-related modules. Phorbol myristate acetate (PMA) was used to induce macrophage modelling in THP-1 cells, and ox-LDL was used to induce macrophage foam cell formation. The experiment was divided into Negative Control (NC) group, Model Control (MC) group, AIM2 overexpression + ox-LDL (OE AIM2 + ox-LDL) group, and AIM2 short hairpin RNA + ox-LDL (sh AIM2 + ox-LDL) group. The intracellular cholesterol efflux rate was detected by scintillation counting; high-performance liquid chromatography (HPLC) was used to detect intracellular cholesterol levels; apoptosis levels were detected by TUNEL kit; levels of inflammatory markers (IL-1β, IL-18, ROS, and GSH) were detected by ELISA kits; and levels of AIM2 and ABCA1 proteins were detected by Western blot. Bioinformatic analysis revealed that the turquoise module correlated most strongly with AS, and AIM2 and ABCA1 were co-expressed in the turquoise module with a trend towards negative correlation. In vitro experiments demonstrated that AIM2 inhibited macrophage cholesterol efflux, resulting in increased intracellular cholesterol levels and foam cell formation. Moreover, AIM2 had a synergistic effect with ox-LDL, exacerbating macrophage oxidative stress and inflammatory response. Silencing AIM2 ameliorated the above conditions. Furthermore, the protein expression levels of AIM2 and ABCA1 were consistent with the bioinformatic analysis, showing a negative correlation. AIM2 inhibits ABCA1 expression, causing abnormal cholesterol metabolism in macrophages and ultimately leading to foam cell formation. Inhibiting AIM2 may reverse this process. Overall, our study suggests that AIM2 is a reliable anti-inflammatory therapeutic target for AS. Inhibiting AIM2 expression may reduce foam cell formation and, consequently, inhibit the progression of AS plaques.

## Introduction

According to survey data published in “The Lancet” in 2020, atherosclerosis (AS) affects the health of over 523 million people worldwide^1^. Ischemic heart disease caused by AS resulted in approximately 12 million deaths globally in 2019^2^. Consequently, addressing the health crisis posed by AS is a challenge that cardiovascular physicians worldwide must confront.

A thorough understanding of the pathological mechanisms underlying the development of a disease forms the foundation for effective treatment. Although the pathogenic mechanisms of AS are complex, increased permeability follows endothelial cell injury leads to infiltration and aggregation of inflammatory cells (macrophages, lymphocytes) in the vascular intima occur, which are transformed into foam cells after phagocytosis of lipids. The accumulation of foam cells leads to the formation of lipid stripes and eventually lipid plaques, representing a crucial process in the occurrence of AS^3^. In this process, the foaming of inflammatory cells, particularly macrophages, after lipid was engulfed, is a pivotal step. Evidence suggests that macrophage-derived foam cells are the main source of foam cells in AS lesions^4^. Therefore, inhibiting the foaming of macrophages has become a current research focus in AS treatment.

Reverse cholesterol transport (RCT) refers to the process where high-density lipoprotein (HDL), acting as a receptor, transports cholesterol from extracellular cells, including macrophages, to the liver for breakdown and metabolism. Subsequently, the cholesterol is excreted from the body through the intestines. The normal RCT function of macrophages is crucial in preventing their foaming effectively^5^.

Recent reports have highlighted that the core protein of RCT: ATP-binding cassette transporter A1 (ABCA1), is continuously suppressed in the pathological process of AS. Additionally, the expression level of ABCA1 is negatively correlated with plaque progression^6,7^. Conversely, two other studies suggest that the AIM2 is highly expressed in AS plaques and positively correlates with plaque progression^8,9^. Therefore, the potential connection between ABCA1 and AIM2 has sparked our significant interest. To address this, we conducted bioinformatic analysis and in vitro experiments, aiming to unravel this question. This connection could be a key factor in macrophage foaming and might offer a new perspective for AS treatment.

## Materials and methods

### Bioinformatics

#### Data source and processing

Expression profiles and sample information from three datasets, GSE43292, GSE100927, and GSE24495, were downloaded from the Gene Expression Omnibus (GEO, https://www.ncbi.nlm.nih.gov/geo/) database. These datasets were annotated using the GPL6244 [HuGene-1_0-st] Affymetrix Human Gene 1.0 ST Array, GPL17077 Agilent-039494 SurePrint G3 Human GE v2 8 × 60 K Microarray 039381, and GPL10687 Rosetta/Merck Human RSTA Affymetrix 1.0 microarray platforms, respectively.

The GSE43292 collected 64 arterial intima samples, including 32 macroscopically intact tissue and 32 atheroma plaques, from 32 atherosclerosis patients. The GSE100927 included 104 peripheral artery samples, with atherosclerosis and control samples derived from peripheral arteries in the neck, femur, and below the knee region. This dataset comprised 44 control samples and 61 atherosclerosis samples. The GSE24495 included 113 carotid artery plaque samples from atherosclerosis patients. Since GSE100927 contained peripheral artery samples from the femur and below the knee region, only the carotid artery data was used in subsequent experiments (Table 1).

**Table 1:** Dataset Information.

Data set	Number of control group samples	Number of plaque samples	Total sample size	Histogenesis
GSE43292	32	32	64	Carotid artery
GSE100927	12	29	41	Carotid artery
GSE24495	0	113	113	Carotid artery

The bioconductor package in the R version 4.2.2 software was employed for background correction, normalization, and expression value calculation. The limma package in the R version 4.2.2 software was utilized to calculate differentially expressed genes (DEGs) between two groups, with a set threshold of adjusted P-value < 0.05 and expression fold change ≥ 2 (| log2FC |≥ 1.00) for DEG selection.

#### Weighted gene co-expression network analysis (WGCNA)

The expression matrix data of AS-DEGs was used to construct a POP-WGCNA network by the WGCNA package in the R version 4.2.2 software. The optimal soft-thresholding power (β) for creating a scale-free network was determined through the unscaled network approach. Subsequently, the adjacency matrix was generated by raising β to the power, and a topological overlap matrix was created using β exponentiation. A hierarchical clustering tree was then built with the topological overlap matrix as the basic element. Module division, merging, and gene dendrogram plotting were performed using the dynamic hybrid cut method.

After module division, characteristic vectors (module eigengenes, ME) for each module were computed. These MEs were correlated with clinical traits of AS patients and normal individuals. The Pearson correlation coefficient was used to assess the correlation between MEs and the clinical traits of the samples, defining the module with the highest correlation with AS as the hub module.

#### Correlation test

The Pearson correlation test completed the correlation analysis of the hub genes. The heat map drawing of the correlation between hub genes was completed by using the corrplot package in the R version 4.2.2 software. At the same time, Spearman correlation analysis and multiple linear regression analysis were performed on the expression of differentially expressed genes in GSE43292、GSE100927 and GSE24495.

### In Vitro* experiments*

#### Cell culture

THP-1 cells were supplied by Procell Life Science&Technology Co., Ltd. (Wuhan, China) and cultured in RPMI-1640 medium (containing 10% FBS, 1% P/S) (Procell, Wuhan, China) at 37 ℃ and 5% CO_2_. THP-1 cells were stimulated with 100 ng/mL of phorbol myristate acetate (PMA) (Absin, Shanghai, China) for 72 h to induce their differentiation into macrophages, and the cell morphology was observed under electron microscope (Hitachi, Japan).

#### Cell transfection and grouping

To manipulate AIM2 expression in THP-1 macrophages, we transfected the cells with shRNA plasmids vectors and overexpression plasmids vectors targeting the expression of AIM2. And blank plasmids vectors were used to transduce THP-1 macrophages as a control group. All vectors were constructed by Chongqing Yitel Bioengineering Co., Ltd. (Chongqing, China). The THP-1 macrophages at 1× 10^6^ cells/mL were inoculated in 6-well plates, and the plasmid vectors were transfected into the cells for 24 h according to the manufacturer’s protocol. To test the transfection efficiency, the supernatant was replaced with fresh RPMI-1640 medium, and the cells were incubated for another 1 h. The transfection efficiency was evaluated by performing real-time quantitative polymerase chain reaction (RT-qPCR) to detect AIM2 expression.

Cells were treated with oxidized low-density lipoprotein (ox-LDL, Solarbio, Beijing, China)^10^. According to the different treatments, THP-1 macrophages were divided into negative control (NC) group, model control (MC) group, overexpression AIM2 + ox-LDL (OE AIM2 + ox-LDL) group, and short hairpin RNA AIM2 + ox-LDL (sh AIM2 + ox-LDL) group. Except for the NC group, the other three groups were stimulated 4 h by adding 150 μg/mL ox-LDL to the cells after discarding the culture medium. Subsequently, replaced with fresh RPMI 1640 medium culture for 24 h. Then the cells were treated with 5 mM ATP(Beyotime Biotechnology,Shanghai, China) for 30 min and rinsed with PBS. The NC group was always cultured in fresh RPMI-1640 medium.

#### RNA isolation and RT-qPCR

As described previously, total RNA was extracted and quantified by Trelief^®^ RNAprep FastPure (TsingkeBiotechnologyCo., Ltd. Beijing, China). Then, Goldenstar^™^ RT6 cDNA Synthesis Kit Ver.2 (Tsingke, Beijing, China) was used to synthesise RNA into cDNA; The TSE202 2 × T5 Fast qPCR Mix (SYBR Green I) (Tsingke, Beijing, China) was used to amplify the AIM2 gene, and it was quantified by real-time fluorescence quantitative PCR instrument (Bio-Rad, US). GAPDH was used as an internal reference gene, and the relative expression level of AIM2 gene was calculated according to the 2^−ΔΔCt^ formula. AIM2 forward primer sequence: 5′-TCCACCCTCATGGGACCTGTAT-3′, AIM2 reverse primer sequence: 5′-TGGCAT- TTCGGGGCAACAT-3′. GAPDH forward primer sequence: 5'-AGGTCGGTGTGAACGGATTTG-3', reverse primer sequence: 5'-TGTAGACCATGTAGTTGAGGTCA-3'.

#### Detection of cholesterol efflux rate

The THP-1 macrophages of 1 × 10^6^ cells/mL were inoculated into 6-well plates. Except for the NC group, the other three groups were incubated for 24 h with RPMI 1640 containing 0.2 μCi/mL 3H cholesterol after stimulating the cells with 150 μg/mL ox-LDL for 4 h. Subsequently, the cells were washed twice with PBS and balanced for 24 h by adding fresh RPMI-1640 medium. The supernatant was discarded and given 5 mM ATP to treat the cells for 30 min. Following the instructions of the total cholesterol assay kit (Nanjing Jiancheng bioengineering Institute, China) the radioactivity of 3H cholesterol in the cell supernatant and cell lysate was detected by liquid scintillation counting, respectively. The cellular cholesterol efflux rate was calculated according to the formula.

#### Detection of intracellular cholesterol levels

The levels of free cholesterol (FC), total cholesterol (TC) and cholesteryl esters (CE) in the cells were detected using High-performance liquid chromatography (HPLC) analysis. Briefly, the cells were washed twice with PBS, then 100 μL of TritonX-100 was added to fully lysed the cells, which were then mixed with 10 μL of enzyme reaction buffer and divided into two portions, followed by the addition of cholesterol oxidase and cholesterol esterase, respectively; Finally, 100 μL of methanol-ethanol (1:1) was added to terminate the reaction and the supernatant was collected to measure FC and TC. The CE is equal to the TC minus the FC.

#### Detection of apoptosis level

The cells were cultured in round coverslip, and the cells were grouped and treated as before. Before adding each reagent, the cells were rinsed with PBS for 3 times. The cells were fixed by adding 100μL of 4% paraformaldehyde fixative (Servicebio, Wuhan, China); followed by 100μL of 0.3% (v/v) Triton X-100 incubated for 5 min to permeabilize the cell membrane; then 50μL of TdT buffer (recombinant TdT enzyme: TMR-5-dUTP labelling mixture: equilibrium buffer = 1 μL: 5 μL: 50 μL (1:5:50)) was added to incubate for 10 min; subsequently, the cells’ nuclei were stained with DAPI for 5 min; and finally, the cells were blocked by using anti-fluorescence quencher. Confocal microscopy (HITACHI, Japan) was used to observe the apoptosis of cells in each group.

#### Western blot analysis

After rinsing the cells twice with PBS, RIPA lysis solution (containing PMSF and the protease inhibitor cocktail, Beyotime Biotechnology) was added and the cells were placed on ice for 10 min to fully lyse, and then the cell suspensions were centrifuged at 13,000 rpm for 20 min at 4 °C and collected supernatants. Protein quantification was performed with the BCA Protein Assay Kit (Beyotime Biotechnology). The proteins were mixed with SDS-PAGE protein uploading buffer (5 ×) (Epizyme, Shanghai, China) at a ratio of 4:1 and placed in a 100℃ dry bath incubator for 5 min to denature the proteins. Proteins were separated on 10% SDS-PAGE gel containing 60 μg of protein per lane. The separated proteins were then transferred to PVDF membranes (Amersham, German) which were closed in 5% skimmed milk for 1 h at room temperature. Then the PVDF membranes were incubated with anti-ABCA1 (abclonal A22125, 1:1000), AIM2 Rabbit pAB (abclonal A3356, 1:1000) and GAPDH (abclonal A 19056, 1:1000) at 4 °C overnight. The PVDF membranes were washed 3 times with TBST, followed by incubation with HRP-conjugated secondary antibody (Servicebio G1213,1:20000) for 1 h at room temperature. The PVDF membranes were rinsed 3 times with TBST, and then the immunoreactive bands were visualized on a nucleic acid protein gel imager (Bio-Rad, USA) by using enhanced BeyoECL chemiluminescent reagent (Beyotime Biotechnology).

### Statistics

Statistical analyses were performed using GraphPad Prism version 9.5.1 software. All data are presented as mean ± standard deviation. Independent sample t-tests were employed for data comparison between two groups, and Pearson correlation analysis was utilized for correlation assessment. Fluorescent images were analyzed using Image J version 1.53 k software. A significance level of *p* < 0.05 indicated statistical significance, while *p* < 0. 01 denoted significant differences.

## Results

### Bioinformatics analysis results

#### Co-expression of AIM2 and ABCA1 in AS

**Figure 1:**
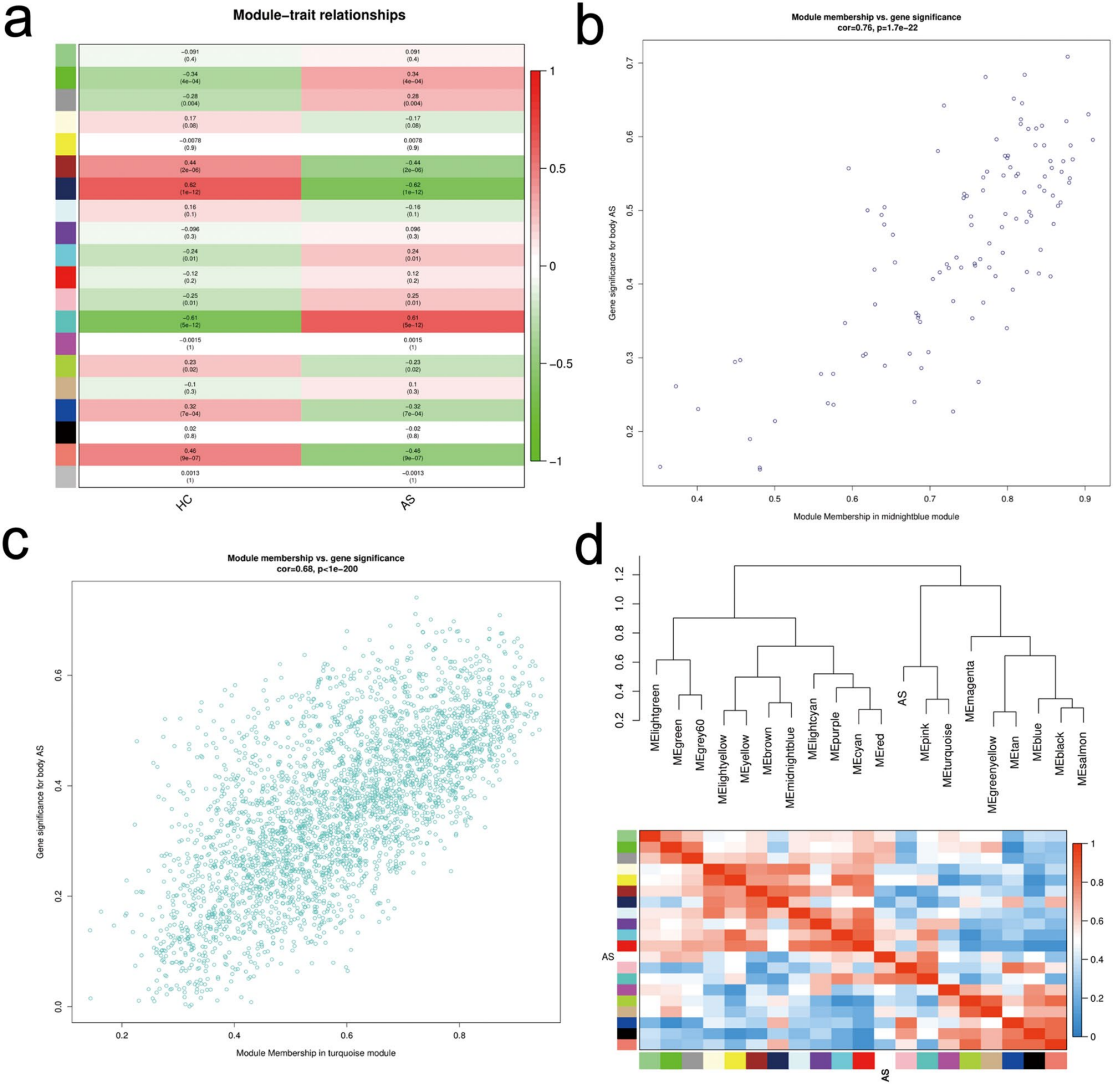
WGCNA analysis. (**a**) The interrelation between modules and AS. (**b**) Module membership in midnight blue module. (**c**) Module membership in turquoise module. (**d**) Module clustering tree.

To validate the co-expression relationship between AIM2 and ABCA1, we constructed a WGCNA for the merged dataset (AS et) from GSE43292 and GSE100927. The results are shown in Fig. 1, where the genes of AS et were assigned to a total of 20 co-expression modules, with the midnightblue module (0.62) and the turquoise module (0.61) being the most strongly associated with the AS phenotype (Fig. 1a).Furthermore, the midnight blue module had a correlation coefficient of 0.76, p-value of 1.7e-22 (Fig. 1b), and the turquoise module had a correlation coefficient of 0.68, p-value less than 1e-200 (Fig. 1c). These results indicate a significant correlation of both modules with the AS phenotype.

Clustering of modules and diseases was performed to screen the core set of genes. turquoise module and AS were clustered under the same subclass (Fig. 1d), suggesting that the genes in the turquoise module are most strongly associated with AS. Subsequently, we found that both AIM2 and ABCA1 belonged to the turquoise module. Therefore, it can be inferred that AIM2 and ABCA1 exhibit a co-expression pattern and are significantly correlated with AS.

#### Correlation in the expression levels of AIM2 and ABCA1

**Figure 2:**
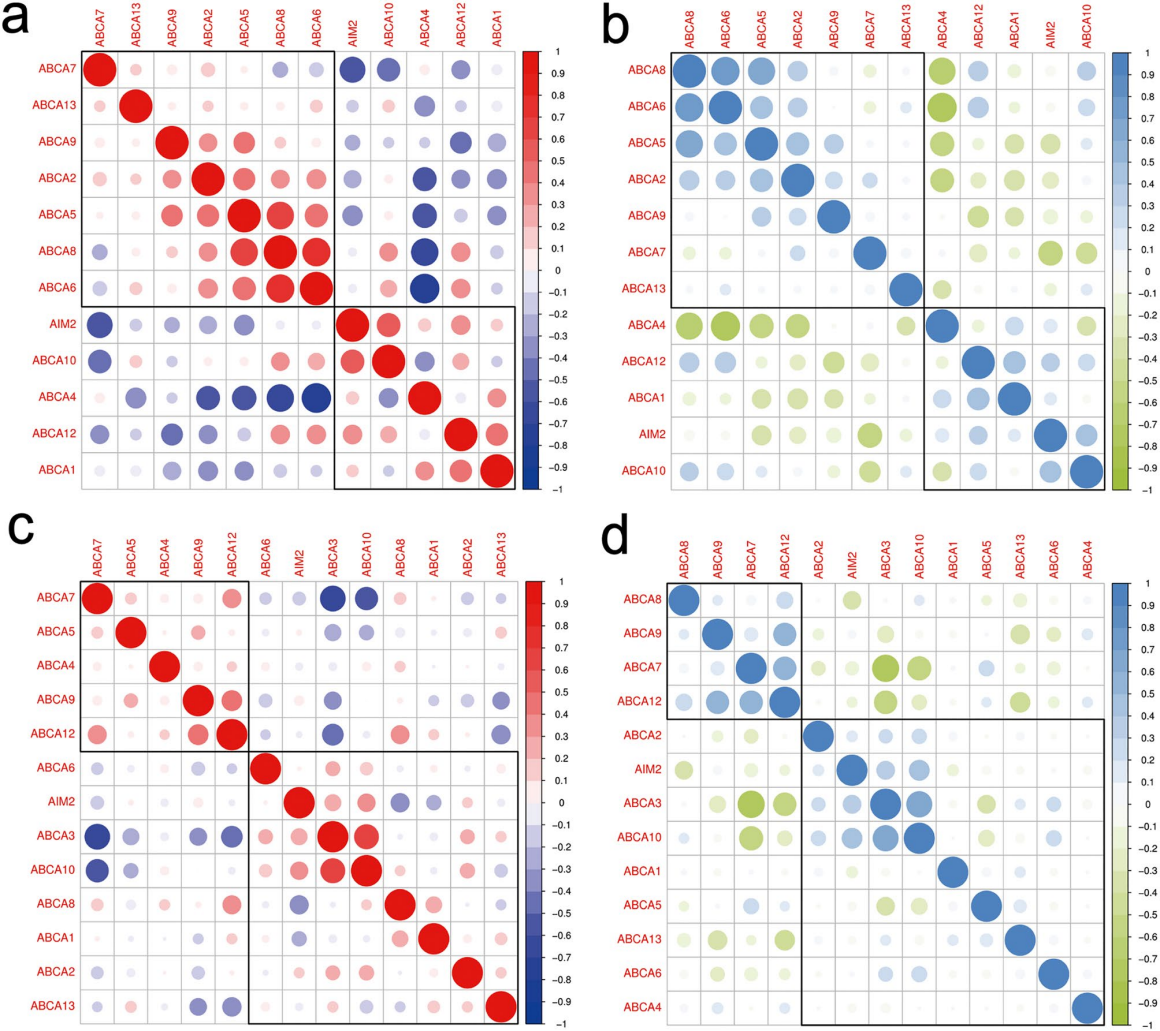
Correlation analysis. (**a**-**b**) Correlation analysis based on AS et dataset. (**c**-**d**) Correlation analysis based on GSE24495 dataset.

To further validate the correlation between AIM2 and ABCA1, we conducted Pearson and Spearman correlation analyses on AIM2 and ABCA family genes in AS et and clustered the genes based on their correlation. The results showed a correlation in the expression of AIM2 and ABCA1 in both the control and AS groups (Fig. 2a and b). To eliminate the potential impact of the control group on the experimental results, we performed the same analysis on GSE24495. The results demonstrated a correlation between AIM2 and ABCA1 in the AS samples alone (Fig. 2c and d).

#### Negative correlation in the expression levels of AIM2 and ABCA1

**Figure 3:**
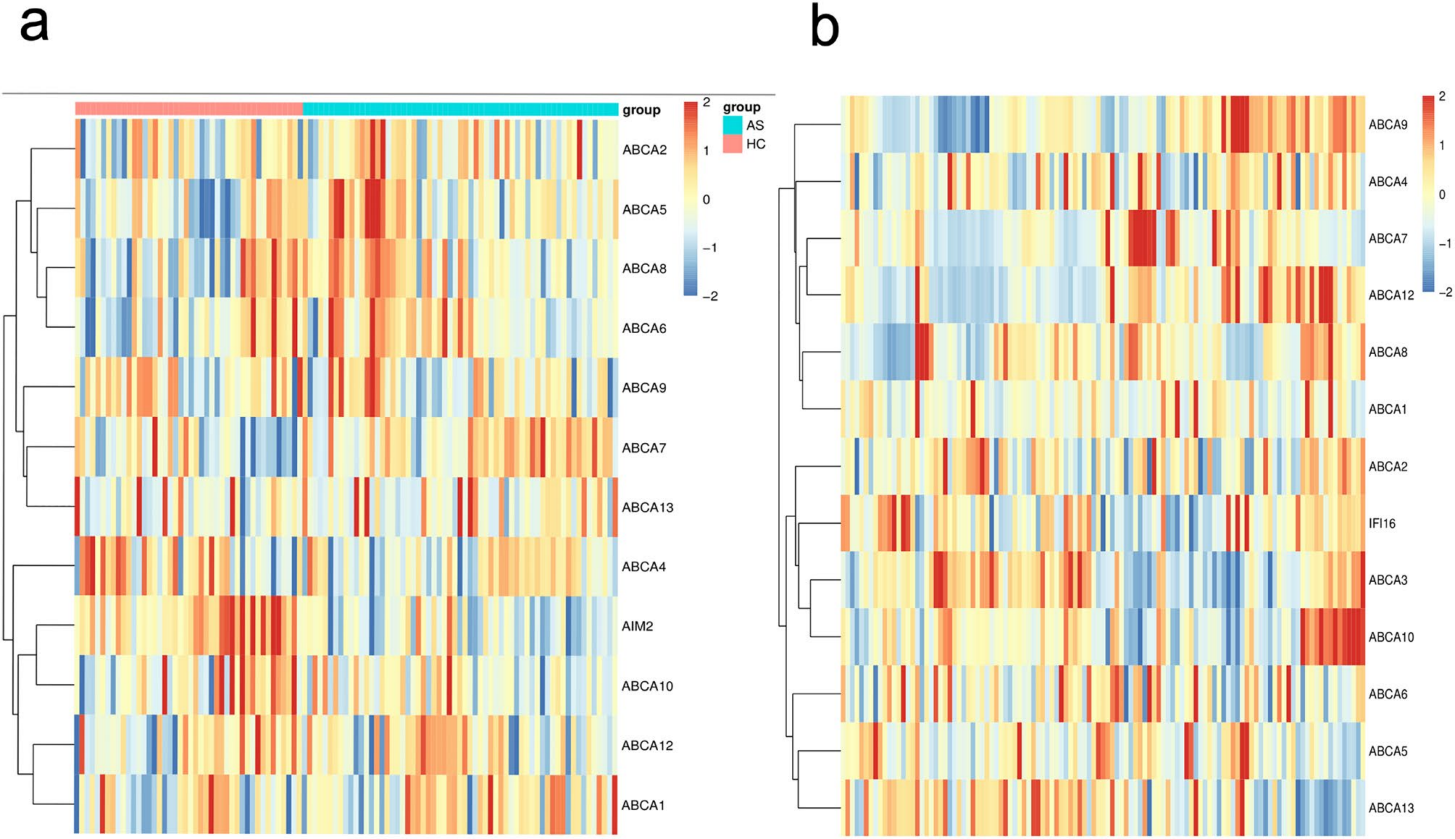
Hierarchical cluster analysis. (**a**) Gene expression heatmap based on HC and AS samples. (**b**) Gene expression heatmap based on HC and AS samples.

To delve into the specific relationship between AIM2 and ABCA1 expression levels in AS, we separately clustered the expression levels of AIM2 and the ABCA family in the AS et and GSE24495 datasets. The results indicate that in both healthy controls (HC) and AS samples, AIM2 and ABCA1 cluster together, suggesting that AIM2 and ABCA1 exhibit correlated differential expression levels in AS samples (Fig. 3a). However, in pure AS samples, AIM2 and ABCA1 did not cluster together. Moreover, the two primary gene clusters showed a trend of negative correlation in expression (Fig. 3b).

### In Vitro* experimental results*

#### Induction of macrophages and foam cells

**Figure 4:**
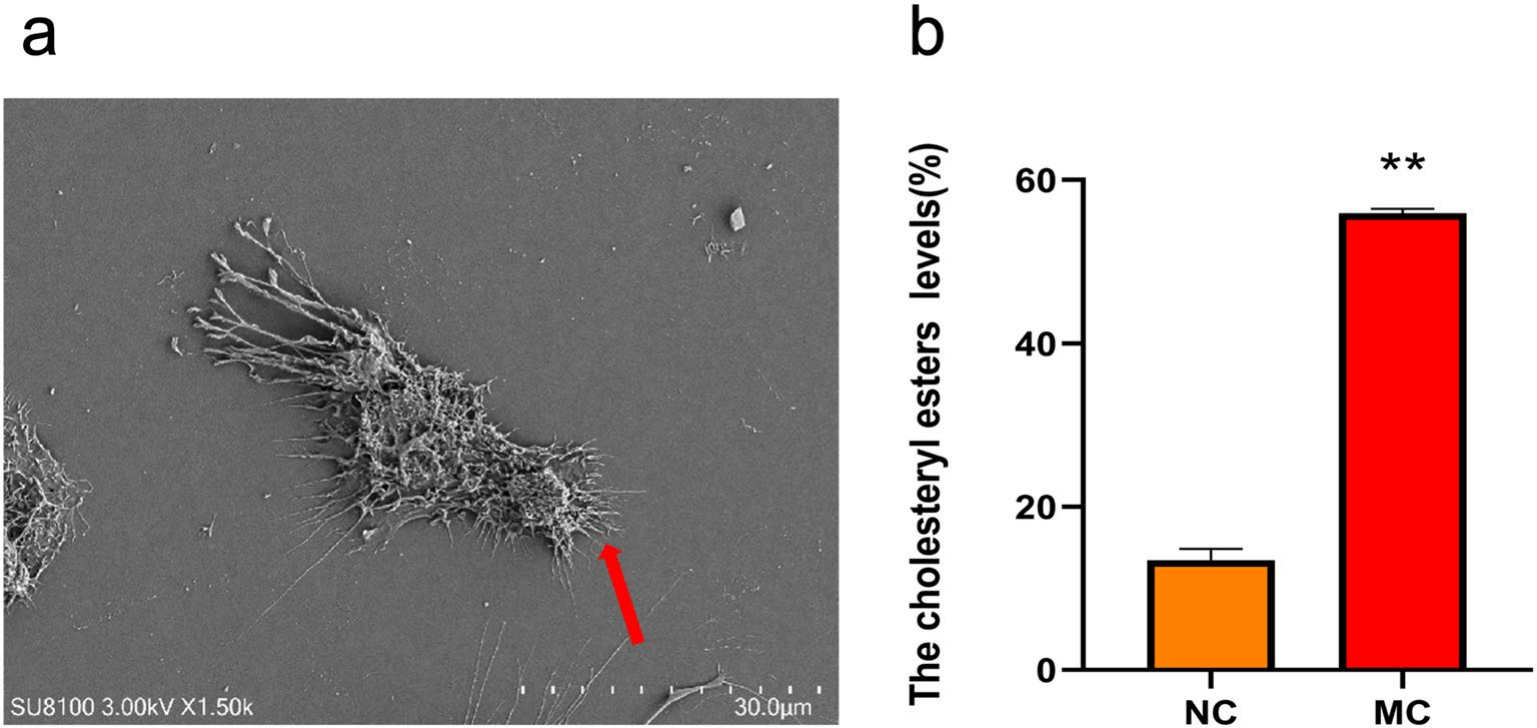
Induction of THP-1 macrophages and foam cell formation. (**a**) transmission electron microscope photo of THP-1 macrophage(Scale, 30.0 μm). (**b**) The level of CE in the NC and MC groups, n = 3, Comparison with NC group, **p < 0.01.

Following PMA induction for 24 h, THP-1 monocytes showed adherence, distortion, and extended pseudopods (as indicated by the arrow in Fig. 4a), indicating that THP-1 monocytes were successfully transformed into THP-1 macrophages. After the ox-LDL stimulated THP-1 macrophages for 4 h, the CE value was higher than 50% in the MC group compared with the NC group, indicating that THP-1 macrophages had been transformed into foam cells (Fig. 4b).

#### Effect of AIM2 gene overexpression/silencing on ox-LDL-induced lipid accumulation in THP-1 macrophages

**Figure 5:**
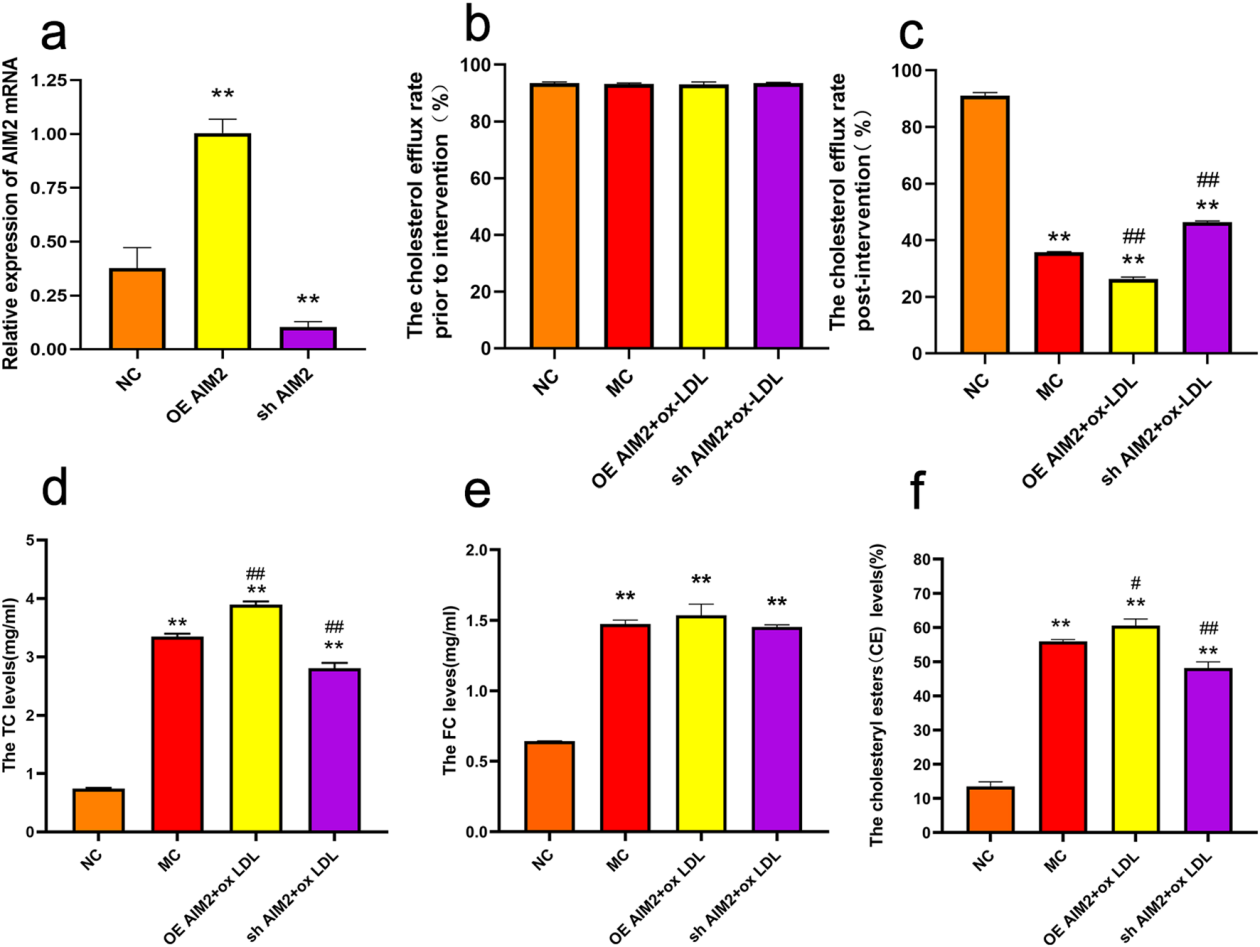
The lipid levels of THP-1 macrophages. (**a**) mRNA expression levels of AIM2 after lentiviral transfection. (**b**-**c**) The Influence of AIM2 on the cholesterol efflux rate in THP-1 macrophages. (**d**-**f**) The Influence of AIM2 on TC, FC and CE levels in THP-1 macrophages. n = 3, Comparison with NC group, **p < 0.01; Comparison with MC group, ##p < 0.01.

We determined whether the AIM2 gene is involved in the process of foam cell formation. The results of lentiviral transfection of THP-1 macrophages showed that AIM2 was effectively silenced in the sh AIM2 group compared to the NC group, whereas the AIM2 mRNA level was significantly increased in the OE AIM2 group (Fig. 5a), indicating that lentiviral transfection was successful. After ox-LDL treated THP-1 macrophages, the cholesterol efflux rate was significantly reduced in the MC group compared to the NC group; AIM2 overexpression exacerbated the inhibition of cholesterol efflux rate in THP-1 macrophages by ox-LDL, whereas this inhibition was significantly alleviated when AIM2 was silenced (Fig. 5b and c). Meanwhile, HPLC analyses showed that FC and TC levels were elevated in THP-1 macrophages after ox-LDL intervention and were influenced by AIM2 levels (Fig. 5d and e). Excessive lipid accumulation in the cells resulted that the CE values were more than 50% in both MC and OE AIM2 + ox-LDL groups, and higher in the OE AIM2 + ox-LDL group (Fig. 5f). All these results suggested that AIM2 aggravated ox-LDL-induced lipid accumulation in THP-1 macrophages, leading to THP-1 macrophage foaminess.

#### Effect of AIM2 gene overexpression/silencing on ox-LDL-induced apoptosis in THP-1 macrophages

**Figure 6:**
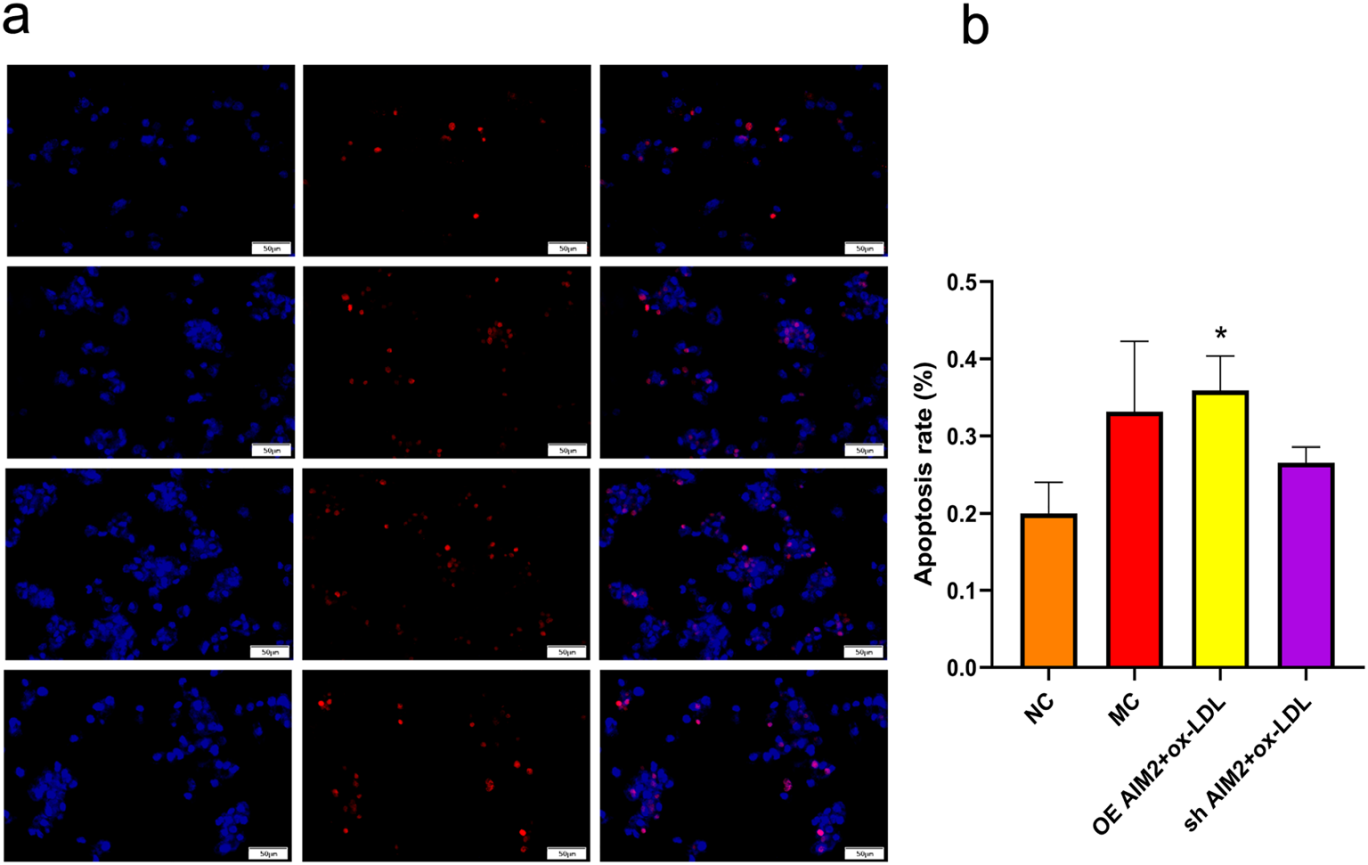
Results of TUNEL detection for cellular apoptosis rate. (**a**) Fluorescent immunostaining image of TUNEL apoptosis, wherein nuclei of positively apoptotic cells emit red fluorescence, and DAPI staining reveals blue fluorescence in the nuclei(Scale, 50 μm). (**b**) Statistical findings of TUNEL in various cell groups post-intervention. n = 3, Comparison with NC group, *p < 0.05.

Macrophage apoptosis is a contributing factor to the accumulation of atherosclerotic damage and AS plaque instability. The apoptosis rate of ox-LDL treated THP-1 macrophages was significantly higher compared with that of the NC group, and there was a tendency for the apoptosis rate to increase in the OE AIM2 + ox-LDL group compared with that of the MC group, and apoptosis in the sh AIM2 + ox-LDL group decreased to some extent, although there was no statistical difference (Fig. 6). These results suggest that inhibition of AIM2 expression ameliorates macrophage apoptosis-induced AS.

#### Effect of AIM2 overexpression/silencing on THP-1 macrophage oxidative stress and inflammatory factor levels induced by ox-LDL

**Figure 7:**
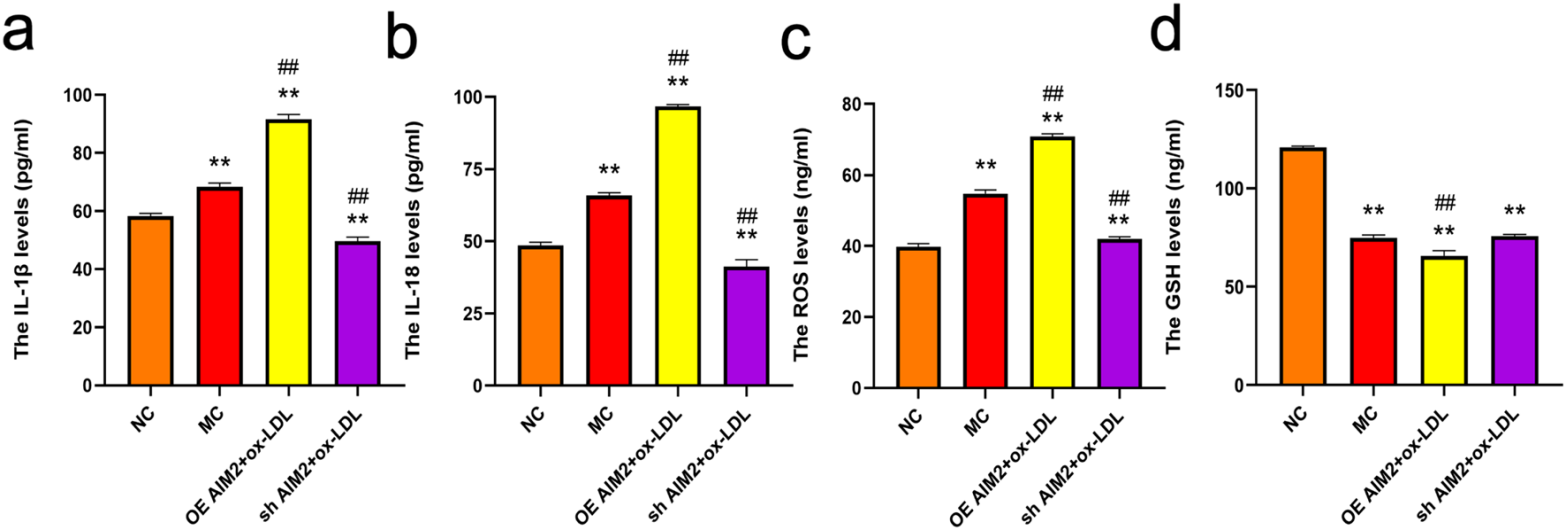
Results of cellular oxidative stress and inflammatory response in each group. (**a**-**b**) The Influence of AIM2 on inflammatory factors IL-1β, IL-18 levels in THP-1 macrophages. (**c**) The Influence of AIM2 on ROS levels in THP-1 macrophages. (**d**) The Influence of AIM2 on GSH levels in THP-1 macrophages. n = 6, Comparison with NC group,**p < 0.01;Comparison with MC group, ##p < 0.01.

AS is a chronic inflammatory disease, and in the inflammatory state macrophages phagocytose ox-LDL and differentiate into foam cells, generating large amounts of reactive oxygen species and inflammatory factors. AIM2 is a known inflammasome, and thus we evaluated the level of oxidative stress and inflammatory factors induced by the AIM2 gene on ox-LDL-induced THP-1 macrophages. Unsurprisingly, MC group cells showed elevated levels of inflammatory factors IL-1β and IL-18 as well as free radicals ROS and suppressed expression of antioxidant enzyme GSH after ox-LDL stimulation. AIM2 overexpression exacerbates the stimulatory effects of ox-LDL on oxidative stress and inflammatory responses. silencing of AIM2 effectively reversed the elevated levels of IL-1β, IL-18, and ROS, and the reduced levels of GSH (Fig. 7).

#### Effect of AIM2 overexpression/silencing on ABCA1 and AIM2 protein expression in ox-LDL-induced THP-1 macrophages

**Figure 8:**
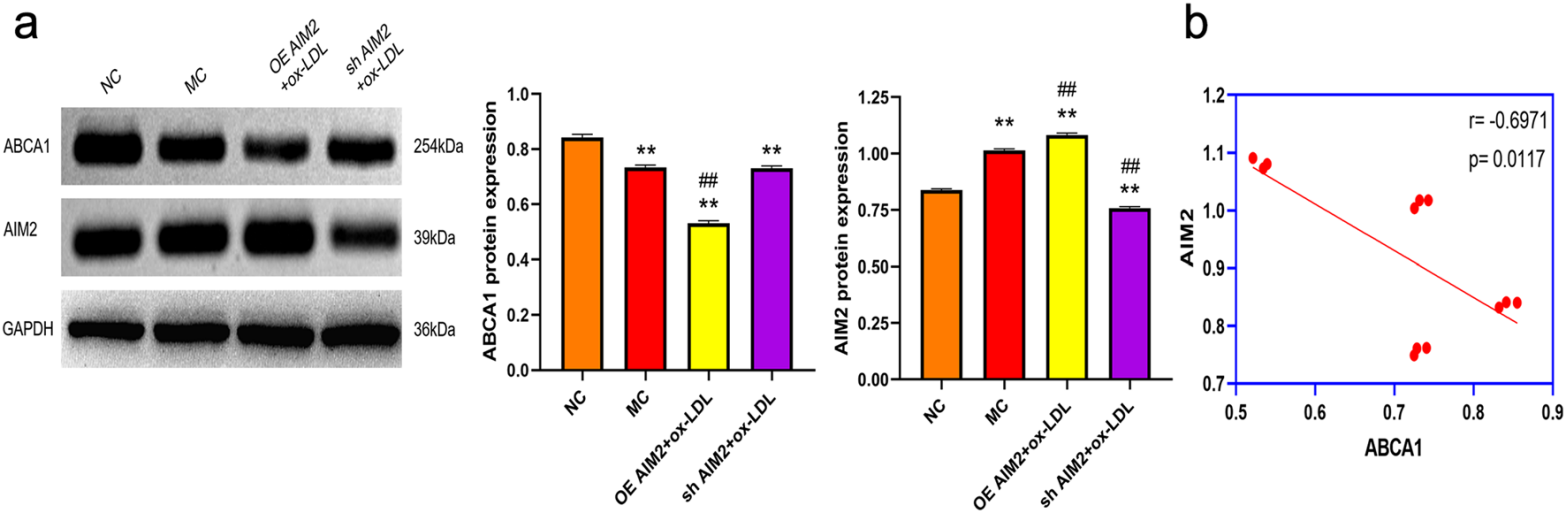
AIM2 is a negative regulator of ABCA1. (**a**) AIM2 and ABCA1 protein expression levels in THP-1 macrophages. (**b**) Correlation analysis of relative protein expression levels of AIM2 and ABCA1. n = 3, Comparison with NC group, **p < 0.01; Comparison with MC group, ##p < 0.01.

We have demonstrated that both AIM2 and ABCA1 are core genes of AS with opposite trends. In addition, high expression of AIM2 inhibits cholesterol efflux rate from THP-1 macrophages. It has been shown that ABCA1, a key molecule in RCT, transports excess cholesterol out of the cell to maintain intracellular cholesterol homeostasis. Therefore, we believe that whether ABCA1 is regulated by AIM2 deserves to be further explored. As shown in Fig. 8a, compared with the NC group, AIM2 protein expression was significantly elevated and ABCA1 protein expression was suppressed in the MC group. Overexpression of AIM2 aggravated the inhibition of ABCA1compared with the MC group; on the contrary, silencing of AIM2 resulted in elevated ABCA1 protein expression. Subsequently, we conducted a correlation analysis between ABCA1 and AIM2, and the results showed that they were significantly negatively correlated (Fig. 8b), which verified the reliability of the results of this postgraduate letter analysis. The above results suggest that AIM2 is an upstream regulator of ABCA1 and that ABCA1 is negatively regulated by AIM2.

## Discussion

Despite the substantial reduction in the associated risk events brought about by antiplatelet and lipid-lowering therapies in AS patients, there still exists other significant residual risks, such as inflammation. Inflammation is considered to play a driving role in the occurrence and development of AS. Similar to the positive effects of antiplatelet and lipid-lowering therapies, inhibiting the inflammatory response has a positive impact on the prognosis of AS patients^11–13^. Therefore, the medical community has been diligently exploring the clinical application of anti-inflammatory treatments for AS patients. However, our understanding of the mechanisms by which inflammation promotes the progression of AS is currently limited, potentially restricting the development of anti-inflammatory interventions. This study addresses this scientific question.

The AIM2 is a pattern recognition receptor with the function of specifically recognizing antigen signals. It identifies and binds cytoplasmic double-stranded DNA (dsDNA) from bacteria, viruses, vaccines, or self-cells^14^. When the HIN-200 domain at the C-terminus of AIM2 recognizes dsDNA, AIM2 recruits the adaptor protein ASC. ASC, as a crucial adaptor molecule, not only contains a PYD domain but also has a CARD domain that directly interacts with Caspase-1. The adaptor protein ASC recruits the effector protein Caspase-1 to the inflammasome complex. The formation of the AIM2 inflammasome brings Caspase-1 molecules close to each other, leading to the autocatalytic activation of Caspase-1^15^. Subsequently, activated Caspase-1 cleaves pro-inflammatory cytokines IL-1β and IL-18 into their mature active forms, promoting the inflammatory response^14,16^.

ABCA1 is a major member of the ABC transporter family, with its human gene located on chromosome 9q31.1, spanning a total length of 149 kb. As a complete membrane transport protein, it consists of two spatially symmetrical transmembrane domains and two nucleotide-binding domains linked together. The latter serves as a specific target for adenosine triphosphate (ATP) binding, providing the energy needed for substance transmembrane transport^17^. ABCA1 exhibits higher levels in inflammatory cells such as macrophages, lymphocytes, and monocytes. The entire process mediated by ABCA1 in the reverse cholesterol transport (RCT) is as follows^18^: firstly, in the initial stage, ATP-driven ABCA1, through specific interaction with apolipoprotein AI, transfers excess cholesterol from macrophages in the arterial wall to the extracellular space, mediating HDL synthesis. Subsequently, cholesterol-enriched HDL is reshaped by phospholipid-cholesterol acyltransferase, forming mature HDL-C. Finally, free HDL-C in the plasma circulates to the liver, where it is recognized and taken up by the type I scavenger receptor, class B, member 1 (SR-BI), expressed on the liver surface. It enters the bile as neutral sterols through liver transport proteins ABCG5/ABCG8 or ABCA1 and is excreted in the feces. Studies have shown that ABCA1-mediated active cholesterol transport accounts for about half of macrophage RCT cholesterol transport^16^. Therefore, the expression level of ABCA1 directly affects the synthesis of blood HDL, inhibits RCT function, promotes lipid deposition in macrophages, and accelerates the formation of atherosclerotic plaques.

Based on our research results and previous literature reports^7,19^, we suspect that ABCA1 is a downstream target of AIM2. Overexpression of AIM2 may promote the expression of downstream inflammatory factors, thereby inhibiting the level of ABCA1, leading to dysfunction of macrophage RCT, causing lipid accumulation in macrophages, and ultimately resulting in macrophage foam cell formation.

As for the increase in AIM2 expression under ox-LDL stimulation, we suspect that it may be related to macrophage oxidative stress. Ox-LDL is a derivative particle of circulating LDL, produced after the oxidation of LDL, and is one of the strong oxidants^20–22^. Moreover, ox-LDL has a strong affinity with the scavenger receptors displayed on the macrophage membrane, making it easier to enter the macrophages and influence the intracellular environment. Among the target organelles of ox-LDL, mitochondria isone of the key factors for AIM2 activation.

Mitochondria is the main sites for producing ATP and is called the “powerhouses” of eukaryotic cells, providing energy for eukaryotic cell activities^23^. The normal life activities of organisms mainly depend on the normal function of mitochondria. Mitochondria participate in ATP synthesis through the tricarboxylic acid cycle, and the Electron Transport Chain (ETC) is the main mechanism for generating energy in mitochondria. ETC is mainly composed of five complexes. Electrons flow between complexes along the electrochemical gradient, with the help of Complex III (ubiquinone-cytochrome c oxidoreductase) and Complex IV (cytochrome c oxidase), and two mobile electron carriers: coenzyme Q10 and cytochrome c^24–27^. In the transfer process, electrons are transferred from the mitochondrial matrix to the inner mitochondrial membrane, generating an electrochemical proton gradient on the inner mitochondrial membrane^28^. Complex V (ATP synthase) depends on this process to oxidize adenosine diphosphate and phosphorylate it into ATP.

Although this process is relatively efficient, about 0.4% to 4% of oxygen cannot be completely reduced and produces the byproduct reactive oxygen species (ROS). Some level of ROS is necessary for maintaining healthy physiological processes and normal cellular functions, and it can be controlled by the cell's normal antioxidant stress mechanism to form a dynamic balance. However, when the exogenous oxidant ox-LDL intervenes, this balance is disrupted. It leads to dysfunction of the ETC, and the impaired ETC promotes the overproduction of ROS, creating a vicious cycle of oxidative stress and respiratory chain defects^29,30^.

Mitochondria are organelles with a double-layered phospholipid membrane structure, where the outer membrane has greater permeability than the inner membrane. This difference in permeability allows the maintenance of the electrochemical proton gradient on both sides of the mitochondrial inner membrane, a necessary condition for maintaining the mitochondrial membrane potential and playing a physiological role, as well as acting as a barrier to prevent the overflow of mitochondrial contents. However, the phospholipid membrane structure of mitochondria is highly susceptible to corrosion by ROS^31^, leading to reduced fluidity and increased permeability. This causes the release of mitochondrial contents into the cytoplasm, including dsDNA. Therefore, combining our ELISA results, we believe that ox-LDL induces oxidative stress in macrophages, increases the permeability of the outer mitochondrial membrane, activates AIM2 through the release of mitochondrial dsDNA, and ultimately leads to decreased ABCA1 expression.

However, this study still has its limitations, we just preliminarily explored the upstream–downstream relationship between AIM2 and ABCA1, but the specific mechanism of action between the two is still unclear and still needs further exploration. In addition, whether AIM2 is able to regulate other genes of the ATP-binding cassette transporter superfamily, such as ABCG1, which can be directly involved in HDL formation. We did not validate the potential link between ABCG1 and AIM2. In subsequent more in-depth studies, we will remedy this deficiency to contribute to a more comprehensive understanding of the supporting mechanisms of RCT and AS.

## Conclusion

AIM2 is a reliable anti-inflammatory therapeutic target against AS. It inhibits ABCA1 expression, leading to abnormal cholesterol metabolism in macrophages and ultimately to foam cell formation. Therefore, inhibition of AIM2 may reverse this process and thus inhibit the progression of AS plaques.

### Supplementary Information

Supplementary Information.

## Data Availability

Data is provided within the manuscript and Supplementary Files.
