# Supplementary figures and images for: Inflammatory corpuscle AIM2 facilitates macrophage foam cell formation by inhibiting cholesterol efflux protein ABCA1
Source: Sci Rep. 2024 May 11;14:10782. doi: 10.1038/s41598-024-61495-4 (PMC11088673; doi:10.1038/s41598-024-61495-4)

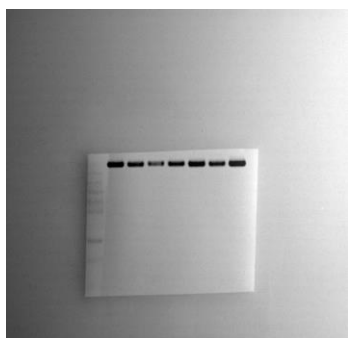

ABCA1-1

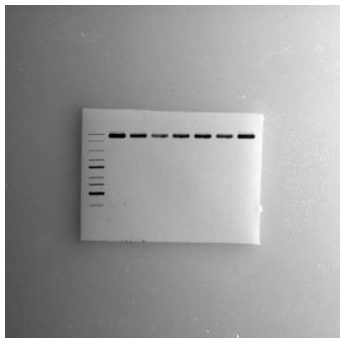

ABCA1-2

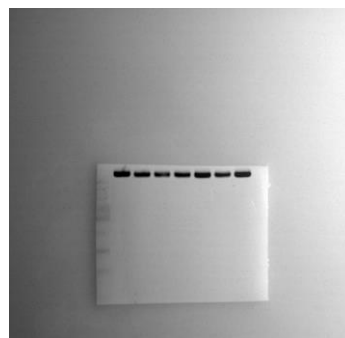

ABCA1-3

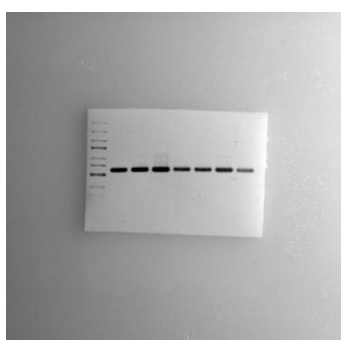

AIM2-1

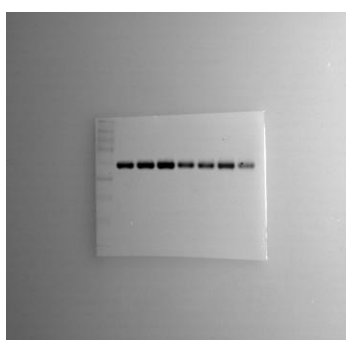

AIM2-2

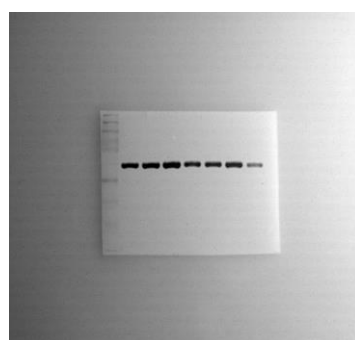

AIM2-3

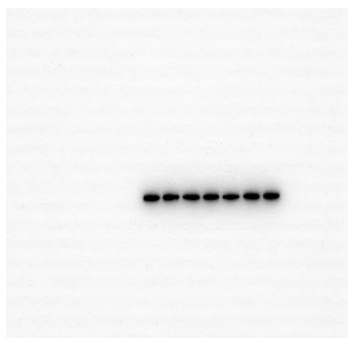

GAPDH-1

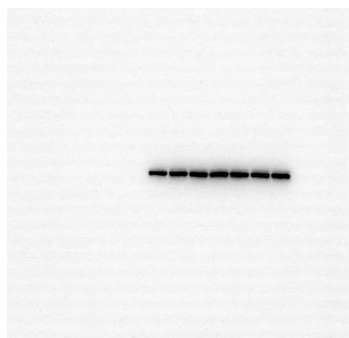

GAPDH-2

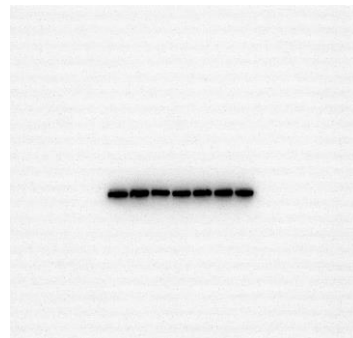

GAPDH-3

Supplement: Supplementary file 1 — Supplementary Information. [file 41598_2024_61495_MOESM1_ESM.pdf]
